# Gitelman syndrome caused by a novel hemiallelic missense mutation in *SLC12A3* revealed by 16q12.2q21 microdeletion

**DOI:** 10.1038/s41439-020-0104-4

**Published:** 2020-05-27

**Authors:** Yuki Abe, Toshiyuki Yamamoto, Yukie Izumita, Shinya Tsukano

**Affiliations:** 1 Department of Pediatrics, Niigata City General Hospital, Niigata, Japan; 2 Institute of Medical Genetics, Tokyo Women’s Medical University, Tokyo, Japan

**Keywords:** Disease genetics, Endocrine system and metabolic diseases

## Abstract

Gitelman syndrome (GS) is caused by biallelic mutations in *SLC12A3* as an autosomal recessive trait. A patient with a de novo 16q12.2q21 microdeletion showed clinical features of GS. *SLC12A3* included in the deletion was analyzed, and a rare missense variant (c.1222A>C [p.N406H]) was identified as hemizygous. Consequently, GS was caused by the revealed *SLC12A3* variant owing to chromosomal microdeletion.

Gitelman syndrome (GS; MIM #263800) is a rare salt-losing tubulopathy characterized by hypokalemic metabolic alkalosis, renal magnesium wasting, and low urinary calcium excretion^1^. Patients with GS may be asymptomatic or have mild symptoms and most commonly manifest muscle weakness. Chronic growth retardation can also be observed. Thus, the age at diagnosis ranges from late childhood to young adulthood.

Biallelic mutations in the solute carrier family 12 (sodium/chloride transporters) member 3 gene (*SLC12A3*), which encodes the thiazide-sensitive NaCl cotransporter, are responsible for GS, indicating an autosomal recessive trait of this condition^2^. Because not all GS patients showed biallelic mutations^3^, mutations in the homologous alleles have never been proven in some cases, and nucleotide alterations in the noncoding regions and genomic copy number aberrations may be unidentified in such cases. Herein, we report a boy with GS caused by a hemiallelic missense mutation in *SLC12A3*, which was revealed by chromosomal deletion.

The male patient was born after 36 weeks of gestation by elective cesarean section. His birth weight and length were 2376 g (mean) and 44.5 cm (10–50th centile), respectively. His parents were healthy and nonconsanguineous. He was admitted to the neonatal intensive care unit and treated with thoracic drainage, medium-chain triglyceride milk, and somatostatin. Afterward, he showed developmental delay and seizure, and some minor anomalies, such as upslanting eyebrows, anteverted nares, and overlapping toes were noted. Chromosomal microarray testing identified a 16q12.2q21 microdeletion (Fig. 1a). The details of this patient have previously been reported^4^.

**Fig. 1:**
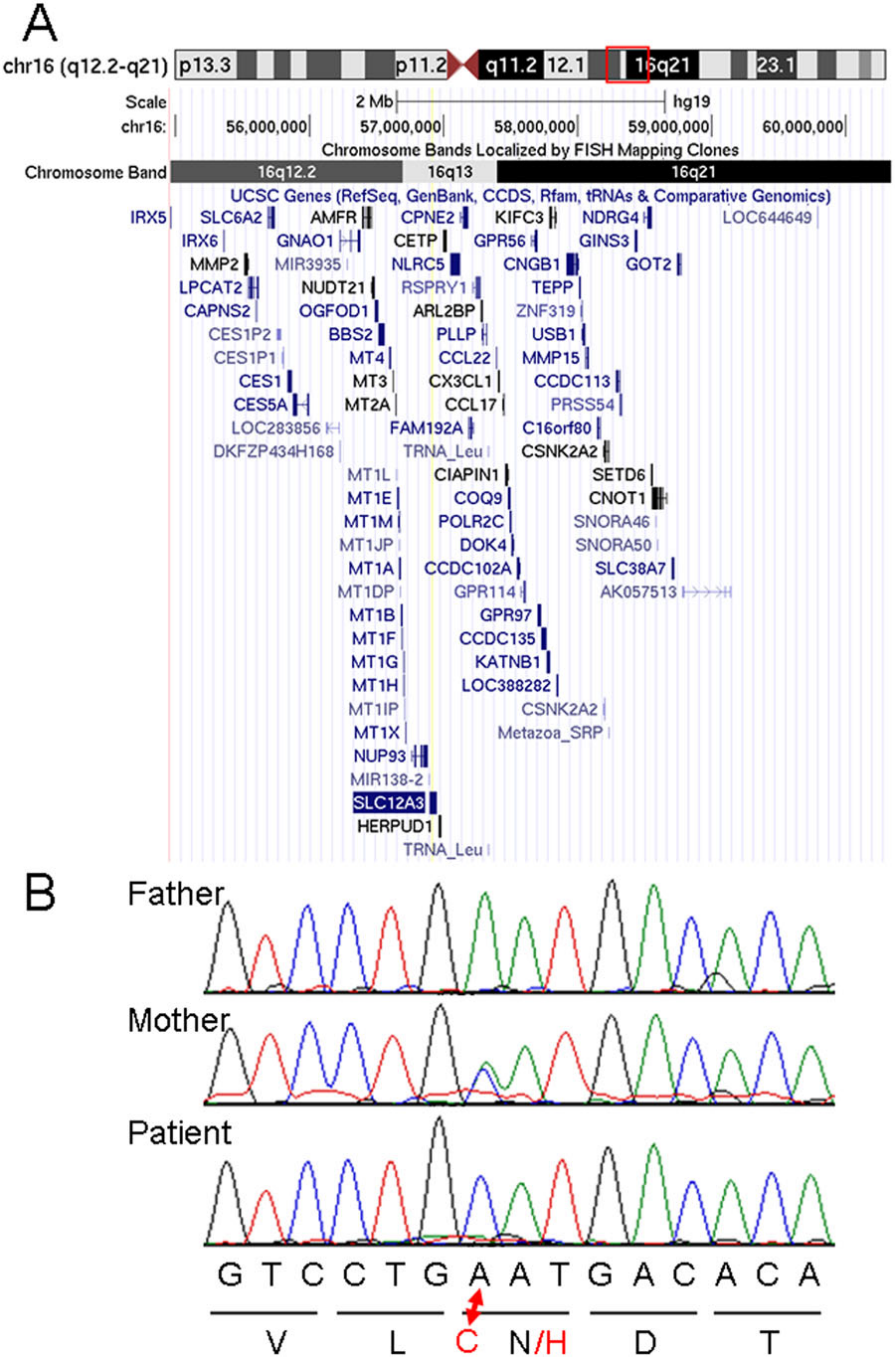
Results of molecular analyses. **a** The genome map around the chromosomal deletion identified in this patient (chr16:54,965,673_60,340,122) captured through UCSC genome browser (https://genome.ucsc.edu/). *SLC12A3*, indicated by blue and white inversion, is included in the deletion region. **b** Electropherograms of the Sanger sequence for the family. Whereas the mother can be diagnosed as a heterozygous carrier of the N406H variant, the patient shows only this variant.

At 8 years of age, his height was 108.9 cm (<3rd centile) and his weight was 16.2 kg (<3rd centile), indicating postnatal growth failure. Routine blood examination revealed hypokalemia with metabolic alkalosis but a normal level of serum magnesium (Table 1). Further examination revealed elevated levels of serum aldosterone (143 pg/mL) and plasma renin activity (16 ng/mL/h). The urinary calcium/creatinine clearance ratio was 0.03. These laboratory findings suggested the existence of tubulopathy in this patient^5^. Because *SLC12A3*, the gene related to GS, is included in the 16q12.2-q21 deletion and GS is an autosomal recessive disorder, we suspected the existence of the *SLC12A3* mutation in the remaining allele.

**Table 1:** Results of blood electrolytes and venous blood gas analysis.

Post treatment (month)		0	2	5	10	12
Potassium supplementation (mEq/kg)		0	2	2.5	2.5	2.5
Na (mEq/L)	(138–145)	137	138	137	139	140
K (mEq/L)	(3.6–4.8)	3.1	3.4	3.4	3.4	3.6
Cl (mEq/L)	(101–108)	99	99	99	99	101
Ca (mg/dL)	(8.8–10.1)	9.6	9.8	9.7	9.8	9.3
IP (mg/dL)	(2.7–4.6)	3.8	3.6	3.8	4.7	4.0
Mg (mg/dL)	(1.8–2.4)	1.8	1.8	1.7	1.7	1.9
pH	(7.35–7.45)	7.435	7.470	7.413	7.395	7.427
HCO_3_ (mmol/L)	(21–27)	27.7	27.1	28.8	29.3	29.7
Base excess (mmol/L)	(−2–2)	3.1	3.4	3.6	3.5	4.6

Values with underlines are indicated as abnormal.

After obtaining written informed consent, nucleotide sequences of all 26 exons and the flanking introns of *SLC12A3* were analyzed using standard Sanger sequencing, and a missense variant, NM_000339.2(SLC12A3):c.1222A>C (p.N406H), was identified as hemizygous (the only minor allele was detected; Fig. 1b) in accordance with our hypothesis. This variant is included in the single nucleotide polymorphism database (dbSNPs: https://www.ncbi.nlm.nih.gov/snp/) with rs759532318.

To confirm parental origins, both parents were also analyzed, and c.1222A>C was identified only in his mother as heterozygous (Fig. 1b). Therefore, the c.1222A>C allele was inherited from this mother, and the 16q12.2q21 microdeletion was suspected to occur on the paternally derived allele as a de novo origin. As a result, the maternally derived c.1222A>C was revealed by paternally derived deletion.

The identified *SLC12A3* variant (p.N406H) is rare, with a global frequency of 0.00009798 and classified as “uncertain significance” in the ClinVar database (https://www.ncbi.nlm.nih.gov/clinvar/). However, most of the prediction scores calculated through wANNOVAR (http://wannovar.wglab.org/) suggested “Damaging”, as shown in Supplementary Table S1. The p.N406H was revealed by a rare event of de novo microdeletion on the homologous allele, as shown by us previously in other clinical conditions^6^.

During the clinical course, serum magnesium levels of this patient often decreased slightly below the lower normal limit (Table 1), which is compatible with the findings in GS^7,8^. Furthermore, after molecular diagnosis, 10 mg potassium gluconate per day (2.5 mEq/kg/day as potassium) was prescribed, and the patient’s serum potassium level was corrected^5^. Then, his body weight gain increased after prescription of oral potassium supplementation from 16.2 kg (−11.3% in obesity degree) to 20.0 kg (−0.6%). For these reasons, we believe that the p.N406H variant identified in this study would be responsible for the clinical features of this patient, and the clinical diagnosis of GS was supported by this molecular diagnosis. According to the database of the National Center for Biotechnology Information (NCBI: https://www.ncbi.nlm.nih.gov/nuccore/NM_000339.2?report=graph), the affected codon 406 is a glycosylation site located in exon 10. Thus, the alteration of asparagine (N) to histidine (H) would prevent normal N-linked glycosylation.

The present patient shows neurological features, including developmental delay and epilepsy, which cannot be explained by GS but would be derived from haploinsufficiency of the genes included in the 16q12.2q21 microdeletion. Although the patient’s weight caught up, his height showed no remarkable improvement (from 108.9 cm [−3.09 SD] to 114.3 cm [−3.04 SD]) after 1 year of potassium supplementation. Thus, his short stature would be caused not only by GS but also by haploinsufficiency of the genes included in the 16q12.2-q21 region.

In conclusion, we presented a patient with GS who developed the disease owing to the monoallelic missense mutation in *SLC12A3*, which was revealed by complete deletion of another allele. Because the 16q12.2q21 microdeletion is of de novo origin, the recurrent risk of GS in this family can be ignored. This information would be beneficial for genetic counseling. Patients with 16q13 deletions should be closely monitored for the onset of GS.

## Supplementary information

Information of the identified variant.

## Data Availability

The relevant data from this Data Report are hosted at the Human Genome Variation Database at 10.6084/m9.figshare.hgv.2853.
