# Supplementary material for: Gitelman syndrome caused by a novel hemiallelic missense mutation in SLC12A3 revealed by 16q12.2q21 microdeletion
Source: Hum Genome Var. 2020 May 27;7:17. doi: 10.1038/s41439-020-0104-4 (PMC7253428; doi:10.1038/s41439-020-0104-4)
Supplement: Supplementary file 1 — Information of the identified variant. [file 41439_2020_104_MOESM1_ESM.pdf]

Supplemental Table S1. Information of the identified variant

Items	Data
Chr	16
Position	56913020
Gene involved	SLC12A3
Nucleotide change	NM_000339.3:c.1216A>C
Amino-acid change	NP_000330.3:p.Asn406His (p.N406H)
Exon	exon10
ExAC_Freq	0.0002
ExAC_EAS	0.0028
gnomAD_exome_ALL	9.80E-05
gnomAD_exome_EAS	0.0014
dbSNP	rs759532318
ClinVar	Uncertain significance
ClinGen	CA8069403
SIFT_score	0.002
SIFT_converted_rankscore	0.721
SIFT_pred	D
Polyphen2_HDIV_score	0.999
Polyphen2_HDIV_rankscore	0.899
Polyphen2_HDIV_pred	D
Polyphen2_HVAR_score	0.946
Polyphen2_HVAR_rankscore	0.832
Polyphen2_HVAR_pred	D
LRT_score	0
LRT_converted_rankscore	0.843
LRT_pred	D
MutationTaster_score	1
MutationTaster_converted_rankscore	0.588
MutationTaster_pred	D
MutationAssessor_score	2.725
MutationAssessor_score_rankscore	0.8
MutationAssessor_pred	M
FATHMM_score	-5.25
FATHMM_converted_rankscore	0.989
FATHMM_pred	D
PROVEAN_score	-3.86
PROVEAN_converted_rankscore	0.724
PROVEAN_pred	D

VEST3_score	0.174
VEST3_rankscore	0.264
MetaSVM_score	-0.04
MetaSVM_rankscore	0.814
MetaSVM_pred	T
MetaLR_score	0.444
MetaLR_rankscore	0.781
MetaLR_pred	T
M-CAP_score	0.142
M-CAP_rankscore	0.825
M-CAP_pred	D
CADD_raw	4.548
CADD_raw_rankscore	0.609
CADD_phred	24.3
DANN_score	0.996
DANN_rankscore	0.759
fathmm-MKL_coding_score	0.984
fathmm-MKL_coding_rankscore	0.824
fathmm-MKL_coding_pred	D
Eigen_coding_or_noncoding	c
Eigen-raw	0.677
Eigen-PC-raw	0.596
GenoCanyon_score	1
GenoCanyon_score_rankscore	0.747
integrated_fitCons_score	0.554
integrated_fitCons_score_rankscore	0.283
integrated_confidence_value	0
GERP++_RS	5.25
GERP++_RS_rankscore	0.731
phyloP100way_vertebrate	8.783
phyloP100way_vertebrate_rankscore	0.914
phyloP20way_mammalian	1.18
phyloP20way_mammalian_rankscore	0.904
phastCons100way_vertebrate	1
phastCons100way_vertebrate_rankscore	0.715
phastCons20way_mammalian	0.98
phastCons20way_mammalian_rankscore	0.49
SiPhy_29way_logOdds	15.136
SiPhy_29way_logOdds_rankscore	0.721
